# Elevations in plasma glucagon are associated with reduced insulin clearance after ingestion of a mixed-macronutrient meal in people with and without type 2 diabetes

**DOI:** 10.1007/s00125-024-06249-7

**Published:** 2024-08-13

**Authors:** Kieran Smith, Guy S. Taylor, Wouter Peeters, Mark Walker, Simone Perazzolo, Naeimeh Atabaki-Pasdar, Kelly A. Bowden Davies, Fredrik Karpe, Leanne Hodson, Emma J. Stevenson, Daniel J. West

**Affiliations:** 1 Oxford Centre for Diabetes, Endocrinology and Metabolism, Churchill Hospital, University of Oxford, Oxford, UK; 2 Human Nutrition and Exercise Research Centre, Population Health Sciences Institute, Newcastle University, Newcastle upon Tyne, UK; 3 School of Biomedical, Nutritional, and Sport Sciences, Newcastle University, Newcastle upon Tyne, UK; 4 Translational and Clinical Research Institute, Newcastle University, Newcastle upon Tyne, UK; 5 Nanomath LLC, Spokane, WA USA; 6 Department of Pharmaceutics, University of Washington, Seattle, WA USA; 7 NIHR Oxford Biomedical Research Centre, Oxford University Hospital Trust, Oxford, UK; 8 Genetic and Molecular Epidemiology Unit, Lund University Diabetes Centre, Department of Clinical Science, Lund University, Malmö, Sweden; 9 Sport and Exercise Sciences, Manchester Metropolitan University, Manchester, UK

**Keywords:** Glucagon, Insulin clearance, Insulin resistance, Obesity, Postprandial glycaemia, Type 2 diabetes

## Abstract

**Aims/hypothesis:**

The temporal suppression of insulin clearance after glucose ingestion is a key determinant of glucose tolerance for people without type 2 diabetes. Whether similar adaptations are observed after the ingestion of a mixed-macronutrient meal is unclear.

**Methods:**

In a secondary analysis of data derived from two randomised, controlled trials, we studied the temporal responses of insulin clearance after the ingestion of a standardised breakfast meal consisting of cereal and milk in lean normoglycaemic individuals (*n*=12; Lean-NGT), normoglycaemic individuals with central obesity (*n*=11; Obese-NGT) and in people with type 2 diabetes (*n*=19). Pre-hepatic insulin secretion rates were determined by the deconvolution of C-peptide, and insulin clearance was calculated using a single-pool model. Insulin sensitivity was measured by an oral minimal model.

**Results:**

There were divergent time course changes in insulin clearance between groups. In the Lean-NGT group, there was an immediate post-meal increase in insulin clearance compared with pre-meal values (*p*<0.05), whereas insulin clearance remained stable at baseline values in Obese-NGT or declined slightly in the type 2 diabetes group (*p*<0.05). The mean AUC for insulin clearance during the test was ~40% lower in the Obese-NGT (1.3 ± 0.4 l min^−1^ m^−2^) and type 2 diabetes (1.4 ± 0.7 l min^−1^ m^−2^) groups compared with Lean-NGT (1.9 ± 0.5 l min^−1^ m^−2^; *p*<0.01), with no difference between the Obese-NGT and type 2 diabetes groups. HOMA-IR and glucagon AUC emerged as predictors of insulin clearance AUC, independent of BMI, age or insulin sensitivity (adjusted *R*^2^=0.670). Individuals with increased glucagon AUC had a 40% reduction in insulin clearance AUC (~ −0.75 l min^−1^ m^−2^; *p*<0.001).

**Conclusions/interpretation:**

The ingestion of a mixed-macronutrient meal augments differing temporal profiles in insulin clearance among individuals without type 2 diabetes, which is associated with HOMA-IR and the secretion of glucagon. Further research investigating the role of hepatic glucagon signalling in postprandial insulin kinetics is warranted.

**Trial registration:**

ISRCTN17563146 and ISRCTN95281775

**Graphical Abstract:**

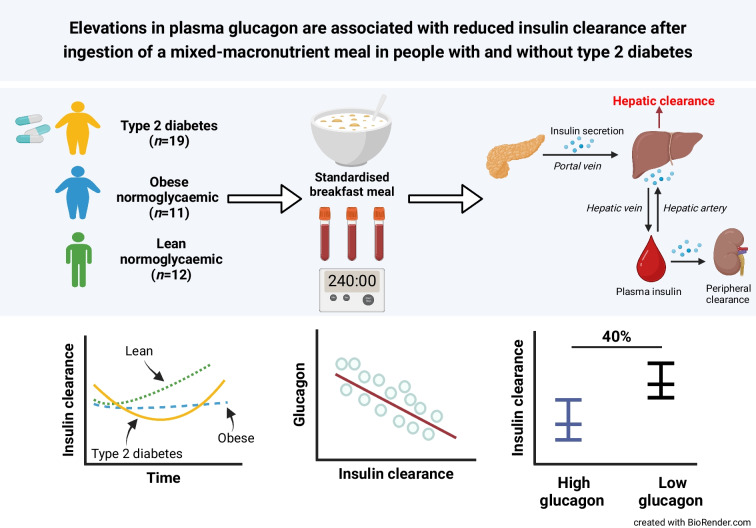

**Supplementary Information:**

The online version of this article (10.1007/s00125-024-06249-7) contains peer-reviewed but unedited supplementary material.


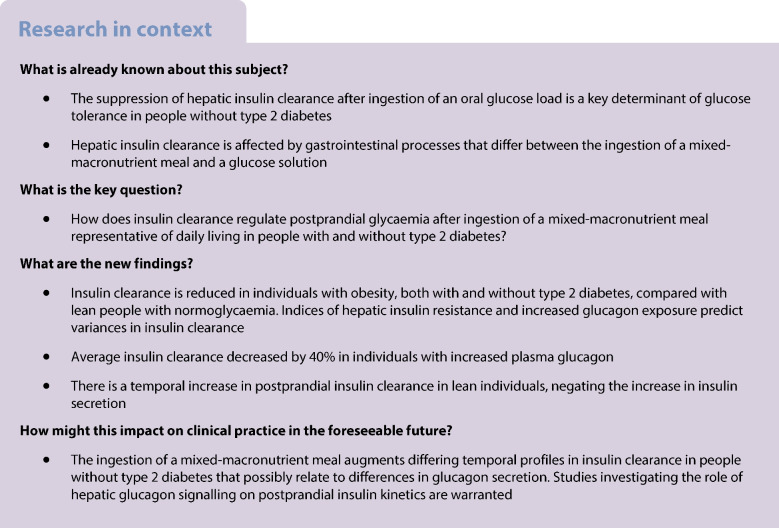


## Introduction

Plasma insulin concentrations reflect the balance between the secretion and clearance of insulin [1, 2]. In healthy individuals, ~70% of newly secreted insulin is extracted by the liver during its first passage through the portal system, which is the major site for insulin clearance [1, 2]. Because of the liver’s capacity to rapidly adjust the insulin clearance rate (ICR), the secretion and hepatic extraction of insulin are tightly related [1]. Hepatic ICR is reduced in individuals with obesity [3–5], central adiposity and hepatic steatosis [6–8]. By enabling a larger fraction of secreted insulin to reach systemic circulation, reduced ICR may serve as an adaptive mechanism to preserve normal glucose tolerance (NGT) in response to insulin resistance [9]. Alterations in ICR may, therefore, precede changes in glucose tolerance [5, 10].

ICR is a dynamic process that changes in response to feeding [9] and macronutrient manipulation [11] on a minute-by-minute basis [1]. Although humans spend most of the day in the fed state [12], few studies have examined the temporal change in ICR in response to meal ingestion. Studies using oral glucose loads have shown there is an immediate and pronounced suppression of ICR in people without type 2 diabetes, which is described as a key determinant of glucose tolerance [3, 9]. However, the use of oral glucose solutions has inherent limitations and does not reflect the hormonal responses or gastrointestinal processes that occur following the consumption of a mixed-macronutrient meal [13, 14]. This requires consideration as ICR may be regulated by the rate of gastric emptying [15] and the secretion of incretin peptides [16] and glucagon [11, 17], both of which are affected by meal type [18] and composition [13, 19]. Whether the adaptive processes reported after the ingestion of glucose [3, 9] are observed after the consumption of a mixed-macronutrient meal remains unknown.

In the current study, we sought to provide insight into postprandial insulin kinetics following the ingestion of a standardised mixed-macronutrient breakfast meal in people with and without type 2 diabetes. We also aimed to identify potential metabolic or hormonal determinants of ICR.

## Methods

### Participants and study design

This study is a secondary analysis of data derived from two randomised, controlled trials investigating the postprandial metabolic effects of protein supplementation in people with [11, 20] and without [21] type 2 diabetes (clinical trial registration no. ISRCTN17563146 and no. ISRCTN95281775). Exclusion criteria for participation included: age >65 years; history of gastrointestinal disease or medication use known to affect gastrointestinal function; BMI >40 kg/m^2^; breakfast skippers; or known dietary intolerances. In addition, participants with type 2 diabetes who were treated with exogenous insulin or glucagon-like peptide 1 (GLP-1) receptor agonists, who had an HbA_1c_ of >80 mmol/mol (9.5%) or who had a duration of type 2 diabetes of <1 year were ineligible. Both trials received ethical approval from the local National Health Service Research Ethics Committee (#18/NE/0372) and from the Research Ethics Committee of the Faculty of Medical Sciences, Newcastle University (#1512/4830/2018). All participants provided written informed consent prior to enrolment.

The study population included 19 individuals with type 2 diabetes (13 men and six women), and 12 lean (Lean-NGT) and 11 centrally obese (Obese-NGT) normoglycaemic adult men (Table 1). In all participants, sex and gender were self-reported. All participants were of white Europid descent (self-reported) and had stable body mass (±1 kg for >2 months). NGT was defined as fasting and postprandial blood glucose concentrations of <5.6 mmol/l and >7.8 mmol/l, respectively, and a return of blood glucose concentrations to preprandial concentrations within 120 min [22, 23]. Participants with type 2 diabetes (HbA_1c_ 56.7 ± 8.8 mmol/mol [7.3 ± 0.8%]) had a median (IQR) duration of diabetes of 4 years (3, 8 years) and were treated by diet and lifestyle modifications (*n*=2), sulfonylurea monotherapy (*n*=1), metformin monotherapy (*n*=5), the combination of metformin with a sulfonylurea (*n*=7) or sodium–glucose cotransporter 2 inhibitor (SGLT2i) (*n*=3), or the combination of metformin, sulfonylurea and a thiazolidinediones (*n*=1). All medications used by the type 2 diabetes group were kept consistent and unaltered throughout; this was to avoid any effects of a missed dose on postprandial glycaemic excursions, which may also affect insulin kinetics [24]. The type 2 diabetes group is representative of the wider English type 2 diabetes population with respect to age, HbA_1c_ and anti-hyperglycaemic medication use [25]. Individuals in the Lean-NGT and Obese-NGT groups were not taking any medication known to affect glucose tolerance or insulin sensitivity.

**Table 1:** Participant anthropometric and metabolic characteristics

Characteristic	Lean-NGT (*n*=12)	Obese-NGT (*n*=11)	Type 2 diabetes (*n*=19)	ANOVA *p* value	Lean-NGT vs Obese-NGT	Lean-NGT vs type 2 diabetes	Obese-NGT vs type 2 diabetes
Women/men (*n*/*n*)	0/12	0/11	6/13	–	–	–	–
Age (years)	36 ± 11	35 ± 7	50 ± 5	<0.001	0.879	<0.001	<0.001
BMI (kg/m^2^)	23.7 ± 1.8	33.7 ± 2.4	32.7 ± 5.7	<0.001	<0.001	<0.001	0.767
Waist (cm)	80.3 ± 6.4	110.8 ± 10.3	105.5 ± 14.7	<0.001	<0.001	<0.001	0.460
Hip (cm)	95.4 ± 4.6	115.2 ± 10.7	107 ± 14.0	<0.001	<0.001	0.021	0.140
WHR	0.84 ± 0.04	0.97 ± 0.07	0.99 ± 0.06	<0.001	<0.001	<0.001	0.601
Fasting biochemistry							
HbA_1c_ (mmol/mol)	–	–	56.7 ± 8.8	–	–	–	–
HbA_1c_ (%)	–	–	7.3 ± 0.8	–	–	–	–
Glucose (mmol/l)	4.4 ± 0.4	4.5 ± 0.3	7.1 ± 1.6	<0.001	0.958	<0.001	<0.001
Insulin (pmol/l)	52.5 ± 20.1	107.6 ± 51.5	86.7 ± 54.1	0.017	0.013	0.133	0.370
ISR (pmol min^−1^ m^−2^)	40.8 (37.2, 54.9)	80.6 (66.7, 112.7)	100.8 (85.8, 119.4)	<0.001^a^	0.009	<0.001	0.938
ICR (l min^−1^ m^−2^)	1.1 ± 1.1	0.8 ± 1.2	1.4 ± 0.4	0.002	0.784	0.017	0.003
Glucagon (pmol/l)	7.0 ± 3.3	12.3 ± 4.6	15.8 ± 6.4	<0.001	0.053	<0.001	0.190
GLP-1 (pmol/l)	17.7 ± 4.9	26.3 ± 12.7	34.1 ± 13.8	<0.001	0.148	<0.001	0.162
GIP (pmol/l)	12.2 ± 7.2	13.4 ± 6.0	15.4 ± 7.8	0.525	–	–	–
Triglycerides (mmol/l)	1.1 (1.0, 1.2)	1.6 (1.2, 2.0)	1.9 (1.0, 3.1)	0.017^a^	0.056	0.025	1.00
NEFA (µmol/l)	508 ± 199	558 ± 196	731 ± 275	0.030	0.868	0.037	0.142
GGT (U/l)	19.6 (13.6, 27.1)	40.3 (18.8, 55.2)	34.8 (27.4, 48.5)	0.010^a^	0.046	0.013	1.00
AST (U/l)	25.2 (19.8, 34.5)	22.5 (20.6, 32.1)	24.0 (18.5, 33.4)	0.479	–	–	–
HOMA-IR	1.8 (1.1, 2.3)	3.3 (2.2, 4.5)	3.9 (2.1, 5.9)	0.002^a^	0.015	0.002	1.00
Adipo-IR	26.2 ± 13.5	61.8 ± 43.4	60.8 ± 38.2	0.010	0.025	0.015	0.986

Continuous data are presented as mean ± SD or median (IQR), whereas categorical data are presented as *n*

Dashes represent data that are not available

All *p* values were adjusted for multiple comparisons

^a^Data were analysed by a Kruskal–Wallis *H* test

AST, aspartate aminotransferase; GGT, γ-glutamyltransferase

### Experimental protocol

Participants arrived via pre-arranged transport to the research facilities after a ~12 h overnight fast. Once rested, participants consumed a breakfast meal consisting of cereal (Cheerios, Nestle, UK) and whole milk, providing 387 kcal from 58% carbohydrates, 27% fat and 15% protein. Venous blood samples were collected from a cannula placed in a forearm vein at baseline (*t*=0 min) and at *t*=15, 30, 45, 60, 90, 120, 150, 180 and 240 min after ingestion of the meal. Meals were consumed within 10 min, and participants remained seated throughout.

### Blood sampling

For the analysis of aspartate aminotransferase, alanine aminotransferase, γ-glutamyltransferase, C-peptide, insulin, NEFA and triglycerides, blood samples were collected in serum collection tubes. Plasma glucagon, total glucose-dependent insulinotropic polypeptide (GIP) and total GLP-1 were measured from blood samples collected in EDTA tubes containing a protease and dipeptidyl-peptidase IV inhibitor [11]. Collected blood samples were centrifuged at 2500 rev/min at 4°C for 10 min with the corresponding supernatant frozen at −80°C until analysis.

### Analytical methods

Blood glucose concentrations were measured from venous whole blood using the enzymatic–amperometric method (Biosen C_Line, EKF Diagnostics, UK). C-peptide, insulin, glucagon, GIP and GLP-1 were measured by ELISA, as described [11]. Liver enzymes, NEFA and triglycerides were measured by routine clinical chemistry using a benchtop clinical analyser (Daytona^+^, Randox Laboratories, UK). NEFA were measured for 60 min post meal to coincide with their suppression [12]. Due to several participants in the Lean-NGT group demonstrating alanine aminotransferase levels below the limit of detection (<12.8 U/l), these data are not included in the analyses.

### Calculations

AUC was calculated using the trapezoidal method and time-averaged for the duration of interest. Adipocyte insulin resistance (Adipo-IR) was estimated from the product of fasting NEFA and fasting insulin concentrations [9]. The suppression of NEFA (%) was taken as the change in NEFA concentrations from baseline to nadir values divided by baseline, and then multiplied by 100. HOMA-IR, an indirect measurement of insulin resistance (primarily hepatic), was determined from fasting insulin and glucose concentrations. Oral minimal models were used to compute measures of insulin sensitivity (*S*_i_) and beta cell function (ɸ_total_) using MATLAB (version R2022b, MathWorks, USA; www.uk.mathworks.com) and SAAM II software (version 2.3.3, Nanomath, USA; www.nanomath.us) [26].

### Insulin secretion and clearance

Pre-hepatic insulin secretion rates (ISRs) were calculated from the deconvolution of C-peptide concentrations using a two-compartmental model and population-derived metrics of C-peptide kinetics [27]. Basal ICR was calculated as fasting ISR divided by fasting insulin, whereas during the feeding test, postprandial ICR was calculated using a single-pool model [9]. As the clearance of insulin by peripheral tissues is constant over a wide physiological range of insulin concentrations [2], differences in ICR from this model primarily reflect the removal of insulin by the liver. Prior to modelling, plasma insulin concentrations were smoothed by cubic spline fitting, and the derivative of insulin over time was calculated from smoothed data using 5 min intervals. The rates of insulin extraction (*Rd*_ins_) and ICR over a given time interval (*t*) were calculated using:

$$\begin{array}{l}{Rd}_{\mathrm{ins}}(t) =\mathrm{ ISR}(t) - [\mathrm{d}I(t)/\mathrm{d}t]\hspace{0.17em}\times \hspace{0.17em}V\\ \mathrm{ ICR}(t) = {Rd}_{\mathrm{ins}}(t) / I(t)\end{array}$$

where *I* is insulin concentrations at time point (*t*), and *V* is the distribution volume for insulin (141 ml kg^−1^ [28]). The average volume of plasma that was cleared of insulin per min (l min^−1^ m^−2^) during the feeding test was calculated as:

$${\mathrm{ICR AUC}}_{240} = {(\mathrm{AUC }[\mathrm{ISR}(t) / I(t)]}_{240\mathrm{ min}} - [{\mathrm{log}}_{e}({I}_{240\mathrm{ min}}) - {\mathrm{log}}_{e}({I}_{0\mathrm{ min}})]\hspace{0.17em}\times \hspace{0.17em}V) / 240,$$

where *I*_0 min_ and *I*_240 min_ are insulin concentrations at time points *t*=0 min and *t*=240 min, respectively [9]. Total insulin extraction (pmol min^−1^ m^−2^) was calculated as:

$${Rd}_{\mathrm{ins}} {\mathrm{AUC}}_{240} = (\mathrm{ISR }{\mathrm{AUC}}_{240} - [{I}_{240\mathrm{ min}} - {I}_{0\mathrm{ min}}]\hspace{0.17em}\times \hspace{0.17em}V) / 240$$

Hepatic insulin delivery (pmol/min) was estimated as the sum of ISR and plasma insulin concentrations delivered to the liver via arterial circulation; the latter was assumed to be a product of hepatic plasma flow (0.576 l min^−1^ m^2^) and plasma insulin concentrations [28].

### Statistical analysis

A posteriori sample size calculation was performed using previously published data in lean and obese individuals [3]. To detect a 25% difference in ICR AUC with a power of 0.8 and an α of 0.05, it was calculated that ten participants in each group were required.

Normality was assessed using the Shapiro–Wilk test, and non-normally distributed data underwent log_10_ transformation. Differences between means were analysed using a one-way ANOVA for normally distributed data or a Kruskal–Wallis *H* test when the parametric assumption was not met. Corrections for multiple comparisons were performed by applying the Tukey post hoc comparison test for ANOVA or the Dunn–Bonferroni post hoc procedure for the Kruskal–Wallis *H* test. To detect differences in postprandial responses between populations, a two-way, mixed-model ANOVA with Tukey post hoc comparisons was performed. Univariate analyses were performed using Spearman’s ρ test to detect correlations between ICR AUC_240_ and metabolic and hormonal measures. A multivariate linear regression model was used to identify predictors of ICR AUC_240_. Unless stated, all data in the text and in the tables are presented as mean ± SD or as median (IQR) for excessively skewed data. All modelling was performed using MATLAB (version R2022b, MathWorks, USA; www.uk.mathworks.com) and inferential statistics were performed using SPSS Statistics (version 28; IBM, USA; www.ibm.com/spss). Significance was accepted as *p*<0.05, and adjusted *p* values for multiple comparisons are presented.

## Results

### Participant characteristics

Participant characteristics are shown in Table 1. Individuals in the type 2 diabetes group were ~14 years older than the Lean-NGT and Obese-NGT groups (*p*<0.001), but had similar BMI, waist-to-hip ratio and liver enzymes as the Obese-NGT group. Markers of insulin resistance (Adipo-IR and HOMA-IR) were similar between the Obese-NGT and type 2 diabetes groups but were higher compared with Lean-NGT (Table 1).

### Fasting metabolic parameters

Fasting blood glucose concentrations were similar between the respective NGT groups but were elevated in those with type 2 diabetes (*p*<0.001). Fasting plasma insulin concentrations were greater in Obese-NGT vs Lean-NGT (*p*=0.011), which was reflected by the increase in basal ISR (*p*=0.009) since fasting ICR values were similar. Compared with Lean-NGT, basal ISR was greater in the type 2 diabetes group (*p*<0.001), although fasting plasma insulin concentrations were similar, with the latter caused by the increase in fasting ICR in the type 2 diabetes group vs the Lean-NGT group (*p*=0.017) (Table 1). Fasting glucagon concentrations, which are consistent with the presence of hepatic steatosis [29], were increased in the type 2 diabetes group (*p*<0.001) and tended to be greater in Obese-NGT (*p*=0.053) compared with Lean-NGT, with no difference between Obese-NGT vs the type 2 diabetes group (Table 1).

### Postprandial blood glucose and insulin kinetics

**Fig. 1:**
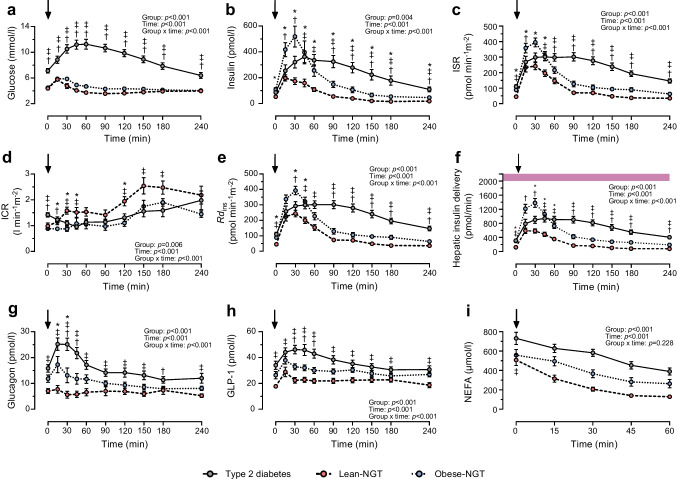
Postprandial glucose (**a**), insulin (**b**), ISR (**c**), ICR (**d**), *Rd*_ins_ (**e**), hepatic insulin delivery (**f**), glucagon (**g**), GLP-1 (**h**) and NEFA (**i**) responses to the ingestion of a mixed-macronutrient breakfast meal in Lean-NGT (red circles, dashed line [*n*=12]) and Obese-NGT (blue circles, dotted line [*n*=11]) individuals, and in people with type 2 diabetes (grey circles, solid line [*n*=19]). The arrow depicts the time of meal ingestion. In (**f**), the purple area represents the maximum hepatic insulin uptake capacity (i.e. ~2000 pmol/min) [2]. In (**g**), glucagon data in the Obese-NGT group are presented as *n*=10. All data are presented as mean ± SEM and were analysed by a two-way, mixed-model ANOVA with Tukey post hoc comparisons. *p* values were adjusted for multiple comparisons: *p<0.05 Lean-NGT vs Obese-NGT; ^†^*p*<0.05 Obese-NGT vs type 2 diabetes; ^‡^*p*<0.05 Lean-NGT vs type 2 diabetes. For clarity, significant main effects for time (i.e. change from baseline) are not presented in the figure

Postprandial glycaemic excursions (AUC_240_) were comparable in Lean-NGT and Obese-NGT but were markedly greater in the type 2 diabetes group (Fig. 1a, Table 2). The change in blood glucose from fasting to peak concentrations was similar in Lean-NGT (1.5 ± 0.9 mmol/l) and Obese-NGT groups (1.7 ± 0.7 mmol/l), but greater in the type 2 diabetes group (4.6 ± 2.1 mmol/l; *p*<0.0001 vs NGT). Both plasma insulin concentrations (Fig. 1b) and ISR (Fig. 1c) were elevated post meal in the type 2 diabetes and Obese-NGT groups, compared with Lean-NGT. While ISR and plasma insulin returned to baseline values within ~90 min post meal in the NGT groups, plasma insulin and ISR excursions were prolonged in the type 2 diabetes group. Compared with Lean-NGT, insulin AUC_240_ was approximately threefold greater in the Obese-NGT and type 2 diabetes groups (*p*<0.01), with no difference between the last two (Table 2). Conversely, overall ISR AUC_240_ was greater in type 2 diabetes compared with both Lean-NGT (*p*<0.001) and Obese-NGT (*p*=0.003), but was not statistically different between Obese-NGT vs Lean-NGT (*p*=0.062).

**Table 2:** Postprandial metabolic responses to the ingestion of a standardised mixed-macronutrient breakfast meal in Lean-NGT and Obese-NGT volunteers and people with type 2 diabetes

Variable	Lean-NGT (*n*=12)	Obese-NGT (*n*=11)	Type 2 diabetes (*n*=19)	ANOVA *p* value	Lean-NGT vs Obese-NGT	Lean-NGT vs type 2 diabetes	Obese-NGT vs type 2 diabetes
Glucose							
Peak (mmol/l)	5.8 ± 0.9	6.2 ± 0.7	11.8 ± 3.3	<0.001	0.932	<0.001	<0.001
AUC_240_ (mmol/l)	4.4 ± 0.4	4.9 ± 0.4	10.0 ± 2.4	<0.001	0.718	<0.001	<0.001
Insulin							
Peak (pmol/l)	230.7 ± 100.1	531.5 ± 271.2	419.9 ± 246.7	0.008	0.007	0.068	0.393
AUC_60_ (pmol/l)	100.2 ± 63.5	270.8 ± 158.5	201.2 ± 128	0.003	0.002	0.031	0.339
AUC_240_ (pmol/l)	69.6 ± 22.8	181.6 ± 97.7	268.2 ± 157.3	<0.001	0.002	<0.001	0.496
ISR							
Peak (pmol min^−1^ m^−2^)	294.8 (179.0, 346.0)	396.3 (328.7, 4749)	339.5 (301.9, 395)	0.029^a^	0.024	0.359	0.472
AUC_60_ (pmol min^−1^ m^−2^)	216.5 ± 60.4	342.0 ± 82.8	305.7 ± 122.5	0.011	0.011	0.048	0.598
AUC_240_ (pmol min^−1^ m^−2^)	100.7 ± 20.6	170.0 ± 42.5	266.4 ± 98.4	<0.001	0.062	<0.001	0.003
GLP-1							
Peak (pmol/l)	26.0 (24.2, 44.8)	38.1 (35.9, 46.6)	47.7 (38.5, 56.6)	0.005^a^	0.600	0.024	0.248
AUC_240_ (pmol/l)	25.3 ± 6.8	33.3 ± 6.8	41.5 ± 14.3	<0.001	0.071	<0.001	0.216
Glucagon^b^							
Peak (pmol/l)	7.9 (7.0, 9.8)	15.2 (11.5, 21.7)	26.6 (21.5, 38.2)	<0.001^a^	0.081	<0.001	0.235
Nadir (pmol/l)	3.6 (1.8, 4.6)	8.2 (4.7, 9.0)	10.0 (4.1, 13.4)	0.005^a^	0.121	0.003	1.00
AUC_240_ (pmol/l)	7.1 ± 4.5	11.0 ± 4.2	16.9 ± 7.4	<0.001	0.053	<0.001	0.097
NEFA							
Nadir (µmol/l)	125.2 ± 40.4	254.1 ± 132.1	382.7 ± 160.9	<0.001	0.007	<0.001	0.026
AUC_60_ (µmol/l)	245.2 ± 80.9	338.5 ± 141.0	561.3 ± 152.4	<0.001	0.036	<0.001	0.004
Suppression (%)	−75.1 (−77.3, −69.9)	−51.4 (−69.3, −45.0)	−49.1 (−66.4, −42.8)	<0.001^a^	0.012	<0.001	0.999
Modelling variables							
ICR AUC_240_ (l min^−1^ m^−2^)	1.9 ± 0.5	1.3 ± 0.4	1.4 ± 0.7	0.014	0.034	0.021	0.989
*Rd*_ins_ AUC_240_ (pmol min^−1^ m^−2^)	91.9 ± 18.4	154.8 ± 38.4	242.0 ± 89.0	<0.001	<0.001	0.012	0.004
Hepatic insulin delivery AUC_240 min_ (pmol/min)	220.0 ± 45.8	506.8 ± 141.5	713.2 ± 316.5	<0.001	0.012	<0.001	0.055
log_10_ *S*_i_	2.5 (2.1, 2.6)	1.4 (1.2, 1.5)	0.6 (0.4, 0.8)	<0.001	<0.001	<0.001	0.177
log_10_ ɸ_total_	2.0 (1.8, 2.3)	2.0 (1.9, 2.1)	1.4 (1.2, 1.5)	<0.001	0.849	<0.001	<0.001

Data are presented as mean ± SD or median (IQR)

All *p* values were adjusted for multiple comparisons

^a^Data were analysed by a Kruskal–Wallis *H* test

^b^Postprandial glucagon data in the Obese-NGT group were available on *n*=10

As shown in Fig. 1d, the postprandial temporal responses in ICR differed between the groups. In Lean-NGT individuals, there was a distinct and immediate increase in ICR from pre-meal values (all *p*<0.05), whereas postprandial ICR remained at baseline values in Obese-NGT. In contrast, there was a modest suppression in ICR at *t*=30 min and *t*=45 min post meal in the type 2 diabetes group (*p*<0.03). Accordingly, ICR was reduced at several points during the postprandial period in the Obese-NGT and type 2 diabetes groups, compared with Lean-NGT (all *p*<0.018; Fig. 1d). Overall, the mean ICR (ICR AUC_240_) was ~35–45% lower in the Obese-NGT and type 2 diabetes groups compared with the Lean-NGT group (*p*=0.034 and *p*=0.021, respectively), with no difference between Obese-NGT and type 2 diabetes groups (Table 2). Compared with Lean-NGT, insulin extraction (*Rd*_ins_ AUC_240_), defined as the average amount of secreted insulin removed per min, was ~40% and ~62% greater in the Obese-NGT and type 2 diabetes groups, respectively (Fig. 1e, Table 2). The increase in *Rd*_ins_ in the Obese-NGT and type 2 diabetes groups was attributable to the greater hepatic delivery of insulin (i.e. during the first and second pass [30]) compared with Lean-NGT (Fig. 1f, Table 2).

### Plasma glucagon and incretin responses

After the meal, glucagon increased in the type 2 diabetes group (*t*=15 min to *t*=45 min; all *p*<0.001), whereas there was no change from fasting values in the NGT groups (Fig. 1g). Compared with Lean-NGT, glucagon AUC_240_ was elevated in type 2 diabetes (*p*<0.001) and appeared greater in Obese-NGT (~55%; *p*=0.053). Glucagon AUC_240_ was similar in the type 2 diabetes and Obese-NGT groups. Similar temporal responses were seen for GLP-1 (Fig. 1h). Accordingly, GLP-1 AUC_240_ was ~63% greater in type 2 diabetes compared with Lean-NGT (*p*<0.001) but was similar between the type 2 diabetes and Obese-NGT groups (Table 2). There was a tendency for GLP-1 AUC_240_ to be elevated in the Obese-NGT group compared with Lean-NGT (~31%; *p*=0.071). GIP was similar between groups (data not shown).

### Postprandial NEFA suppression

In the Lean-NGT group, there was an immediate and significant postprandial decline in NEFA concentrations from fasting values (all *p*<0.01), whereas the post-meal decline in NEFA was delayed in Obese-NGT and type 2 diabetes groups, with statistical reductions from baseline values only being observed from *t*=45 min and *t*=60 min (all *p*<0.01; Fig. 1i). Overall, the absolute suppression of NEFA (%) was ~30% greater in Lean-NGT compared with both Obese-NGT and type 2 diabetes groups (*p*<0.012), with no difference between the Obese-NGT and type 2 diabetes groups (Table 2).

### Insulin sensitivity and beta cell function

There was a stepwise decline in insulin sensitivity (*S*_i_) from Lean-NGT to Obese-NGT to type 2 diabetes (*p*<0.0001). Similar patterns were observed when expressing insulin sensitivity using the oral glucose insulin sensitivity index, whereas insulin sensitivity measured by the Matsuda index was similar between Obese-NGT and type 2 diabetes groups (electronic supplementary material [ESM] Table 1). ɸ_total_ was reduced in the type 2 diabetes group compared with the NGT group (*p*<0.001), consistent with beta cell dysfunction, whereas ɸ_total_ was similar in the Obese-NGT and Lean-NGT groups (Table 2).

### Metabolic associations and predictors of ICR

ICR AUC_240_ was inversely associated with HOMA-IR (*r*=−0.826; *p*<0.001), Adipo-IR (*r*=−0.681; *p*<0.001), glucagon AUC_240_ (*r*=−0.670; *p*<0.001 [*n*=41]) and GLP-1 AUC_240_ (*r*=−0.623; *p*<0.001), as well as waist-to-hip ratio (*r*=−0.525; *p*<0.001). There was also a positive correlation between ICR and *S*_i_ (*r*=0.619; *p*<0.001).

**Fig. 2:**
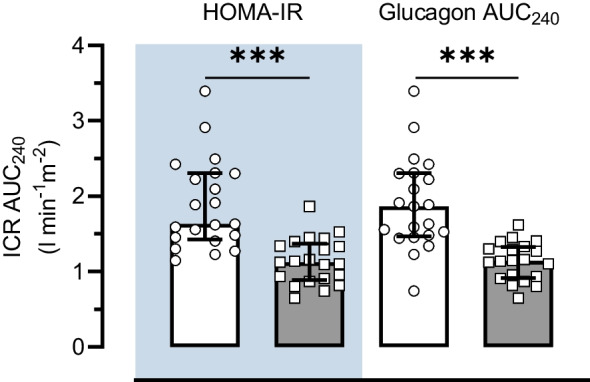
Differences in ICR AUC_240_ in individuals classified as high or low HOMA-IR or glucagon AUC_240_. High (grey bars) and low (white bars) groups were defined as those above or below the group median for HOMA-IR (2.7 AU) or glucagon AUC_240_ (10.7 pmol/l), respectively. Data are presented as median and IQR. ***p<0.001 between low and high groups

Due to one Obese-NGT participant not having available postprandial glucagon data, a multivariate linear regression model was performed on *n*=41 (Table 3). The model demonstrated that log_10_ HOMA-IR and glucagon AUC_240_ were the strongest predictors of log_10_ ICR AUC_240_ in both the unadjusted model (adjusted *R*^2^=0.636; *p*<0.001) and the Adipo-IR-, BMI-, age- and *S*_i_-adjusted model (adjusted *R*^2^=0.670; *p*<0.001). Confirming this, we separated the whole group into high or low based on the group median for HOMA-IR (2.7 arbitrary units [AU]) or glucagon AUC_240_ (10.7 pmol/l). ICR AUC_240 min_ was reduced by 40% (~ −0.75 l min^−1^m^−2^) in the ‘high’ groups compared with the ‘low’ groups (all *p*<0.001; Fig. 2). As elevations in plasma glucagon concentrations are implicated with hepatic steatosis [29], independent of insulin resistance, these results suggest that reduced ICR is a consequence of intra-hepatic lipid accumulation rather than insulin resistance per se.

**Table 3:** Independent predictors of ICR from a multivariate linear regression model

Variable	log_10_ ICR AUC_240_								
	*B*	95% CI *B*		SE	β	*F*	*R*^2^	Adjusted *R*^2^	*p* value
		Lower	Upper						
Model 1						24.424	0.562	0.539	<0.001
Constant	0.305**	0.238	0.372	0.033					
log_10_ HOMA-IR	−0.375**	−0.492	−0.259	0.057	−0.722				
*S*_i_	<0.000	0.000	0.000	0.000	0.095				
Model 2						18.451	0.672	0.636*	<0.001
Constant	0.393**	0.311	0.474	0.040					
log_10_ HOMA-IR	−0.196*	−0.360	−0.033	0.081	−0.377				
*S*_i_	<0.000	0.000	0.000	0.000	−0.083				
Adipo-IR	<0.000	0.000	0.000	0.000	−0.324				
Glucagon AUC_240_	−0.007*	−0.012	−0.001	0.003	−0.300				
Model 3						14.544	0.720	0.670	<0.001
Constant	0.227	−0.453	0.775	0.302					
log_10_ HOMA-IR	−0.255*	−0.429	−0.081	0.086	−0.491				
*S*_i_	<0.000	0.000	0.000	0.000	−0.006				
Adipo-IR	<0.000	0.000	0.000	0.000	−0.243				
Glucagon AUC_240_	−0.008*	−0.013	−0.003	0.003	−0.362				
Age	0.004*	0.001	0.007	0.002	0.251				
BMI	0.001	−0.008	0.009	0.004	0.028				

Data were analysed by multivariate regression model on *n*=41

^*^p<0.05

^**^p<0.01

## Discussion

The temporal suppression of ICR in response to an oral glucose load has been described as a key determinant of glucose tolerance [9]. However, the metabolic handling of a mixed-macronutrient meal differs compared with the consumption of glucose alone [13]. In the present study, we examined postprandial ICR responses after the ingestion of a standardised mixed-macronutrient breakfast meal in Lean-NGT and Obese-NGT individuals, and in people with type 2 diabetes. Our main findings identify a unique temporal variation in ICR that differs between Lean-NGT and Obese-NGT individuals. Specifically, in response to the meal, we observed an immediate increase in ICR in the Lean-NGT group, whereas ICR remained at baseline values in the Obese-NGT group. Although there were differing ICR responses in the NGT groups, overall postprandial glycaemic excursions were similar. Furthermore, we show that overall variances in postprandial ICR are determined by HOMA-IR and elevated plasma glucagon concentrations. Taken together, these findings demonstrate the adaptive responses of ICR to regulate postprandial glycaemia in the presence of insulin resistance.

While our data are in general agreement that ICR is reduced in obese, insulin-resistant individuals [3, 4, 8, 9, 31], this was not reflected by a greater post-meal suppression in ICR in the Obese-NGT group. These findings contrast with what has been reported following an oral glucose load [3, 9]; however, direct comparisons between studies are challenging due to differences in the test meals provided. Indeed, variances in nutrient absorption kinetics [15] and the subsequent islet [17] and incretin [16] responses between meals offer a plausible explanation for the reported variances in temporal ICR. The possible influence of hormonal responses on ICR is suggested by the current data, as shown by the strong inverse associations of GLP-1 AUC_240_ (r=−0.623) and glucagon AUC_240_ (r=−0.670) with ICR AUC_240_. However, our reported post-meal suppression of ICR in the type 2 diabetes group is akin to what is seen after the ingestion of glucose [3, 9]. As our calculated ISRs were twofold greater than what is reported after glucose ingestion [9], this may reflect the inability of the diabetic liver to extract insulin [32], independent of allostatic load (i.e. nutrient or hormonal stimuli). Nevertheless, as we did not perform an oral glucose feeding test, future studies examining the interactions between nutrient composition and postprandial insulin kinetics are warranted.

In our study, we observed a synchronous increase in ISR and ICR in the Lean-NGT group, such that the clearance of insulin overshadowed its secretion, resulting in reduced overall plasma insulin concentrations. Despite this response, overall postprandial glycaemic excursions were minor in Lean-NGT, implying that a proficient insulinaemic response to maintain NGT was achieved. This is consistent with the notion that the balance between insulin secretion and clearance prevents insulin excess [33] or insufficiency [9] and their adverse metabolic consequences. Previous work has demonstrated that during periods of low blood glucose, a temporal increase in ICR negates the increase in postprandial ISR as a possible means to prevent hypoglycaemia [34]. In this regard, there is evidence to support a sensory function of the portal vein that alters the hepatic arteriovenous insulin gradient and hepatic glucose metabolism in response to nutrients and/or hormones [30, 35, 36]. Such a feedback loop may be present here given our observation of a complete post-meal suppression of glucagon in Lean-NGT individuals, which combined with the increase in ISR is suggestive that hepatic glucose production was suppressed [37]. This also offers an explanation for the observed temporal responses of ICR in the Obese-NGT group, for whom glucagon was less suppressed and overall glucagon excursions greater, yet NGT was well maintained. It is, therefore, reasonable to infer that during periods of altered portal nutrient/hormonal flux, the temporal responses in ICR reflect an adaptive mechanism to protect not only against hyperglycaemia [9], but also hypoglycaemia [33].

In the present study, the Obese-NGT and type 2 diabetes groups showed an impaired suppression of NEFA, which is proposed to impair ICR [38]. Mechanistically, increased delivery of NEFA to the liver blunts hepatic insulin signalling and activates peroxisome proliferator-activated receptor α (PPAR-α) [39], which reduces the transcription of carcinoembryonic antigen-related cell adhesion molecule 1 (CEACAM1) [39–41]. CEACAM1 facilitates the internalisation of receptor-bound insulin for endosomal degradation and its specific liver inactivation impairs ICR [40]. Our data suggest that the increased secretion of glucagon may impair ICR, independent of hepatic NEFA delivery. As elevations in plasma glucagon are a metabolic disturbance of hepatic steatosis [29], independent of insulin resistance or glucose tolerance [42], these observations likely describe reduced ICR as a consequence of hepatic steatosis [8]. In support, the infusion of exogenous glucagon was recently shown to directly impair ICR in healthy adults [17]. Indeed, increased hepatic glucagon signalling augments PPAR-α activity [43], which reduces the expression of CEACAM1 [39] and impairs ICR [40]. Of note, glucagon-stimulated PPAR-α activation is augmented further by the presence of fatty acids [44]. These observations may be of interest for the pathophysiology of hepatic steatosis that is described by increased plasma glucagon concentrations [29] and increased hepatic lipid flux [45]; these ‘dual-hit’ signals may offer an explanation as to why CEACAM1 expression [46] and ICR [8, 31] are reduced in people with increased liver fat.

We observed an inverse association between GLP-1 AUC_240_ and ICR AUC_240_, and the substitution of glucagon AUC_240_ with GLP-1 AUC_240_ in our multivariate analyses did not change our findings (see ESM Results; ESM Table 2). As our measurement of plasma glucagon is specific for pancreatic-derived glucagon, this observation is not due to cross-reactivity with the other proglucagon-derived peptide, GLP-1, or ‘gut-glucagon’. Previously, the blockage of the endogenous GLP-1 receptor with exendin(9–39)NH_2_ was also reported to increase ICR [16]. As there are no known GLP-1 receptors in the liver, and exendin-4 shares ~50% amino acid homology with glucagon, this may reflect reduced signalling via the hepatic glucagon receptor.

It has been suggested that the postprandial ICR profile in those with type 2 diabetes is due to the reduced hepatic delivery of insulin (i.e. beta cell dysfunction), rather than an impairment in the liver to adjust the clearance of insulin [3]. In contrast, our data suggest that the impaired suppression of ICR in individuals with type 2 diabetes reflects a maladaptive hepatic response and not a primary beta cell defect. For instance, overall ISR and hepatic insulin delivery were increased in the type 2 diabetes group, but this was not associated with marked deviations in ICR compared with the Obese-NGT group. Thus, although there was a temporal increase in insulin extraction in those with type 2 diabetes, this should not be interpreted that the hepatic handling of insulin is intact or functional. It must be recognised, however, that type 2 diabetes is a heterogenous condition with varying degrees of residual beta cell function. While our data suggest that the impaired suppression of ICR is a primary hepatic defect, this may not be the case for people with more advanced type 2 diabetes and reduced insulin secretory capacity. Moreover, although our reported postprandial ICR profile in the type 2 diabetes group is akin to previous findings in people with type 2 diabetes after drug withdrawal [9], as all anti-hyperglycaemic medications were kept unaltered in our study, we cannot exclude the potential effects of concomitant medication use on postprandial insulin kinetics which may cloud our findings.

We acknowledge that there are further limitations associated with our study which include the secondary, cross-sectional nature of the analyses, the small sample size and the age imbalance between the type 2 diabetes and NGT groups. Furthermore, sex distributions of participants between groups were unmatched in our study. Previous analyses have shown that, although there is an overall reduction in ICR in female individuals compared with male individuals [47], the temporal response in ICR following glucose ingestion between insulin-resistant and insulin-sensitive individuals is qualitatively unaffected by biological sex [3], suggesting that our findings may be generalisable across sexes. Nonetheless, as our sample size did not allow for subgroup analyses stratified by biological sex, future studies examining potential differences in postprandial insulin kinetics in phenotypically matched (age, insulin sensitivity and glucose tolerance) female and male individuals are warranted. As all participants were of white Europid descent, our data also do not reflect the potential ethnic differences in postprandial ICR [28]. In our study, we did not directly quantify hepatic ICR; however, the use of indirect approaches that incorporate insulin and C-peptide kinetic modelling offer an alternative to calculate ICR in vivo during dynamic tests [48]. Moreover, direct measurements of intra-hepatic fat content and insulin sensitivity were not available in our analysis. Although our findings describe a potential link between increased plasma glucagon concentrations and reduced ICR, we were unable to establish causality. As elevations in plasma glucagon are a reported consequence of intra-hepatic fat deposition [29, 42], future prospective studies examining the role of glucagon on ICR in well described cohorts of people with and without hepatic steatosis are warranted.

Previous studies have assessed postprandial differences in ICR in obese and insulin-resistant populations after ingestion of glucose [4, 13] or mixed-macronutrient solutions [13]. Their findings, however, are limited by the reported static indices of ICR (i.e. the ratio of C-peptide/insulin or ISR/insulin AUCs). As demonstrated here, the use of these estimates alone has significant interpretative limitations for the study of postprandial hyperinsulinaemia and masks the dynamic changes in insulin extraction that occur post meal. As failure to appropriately regulate the liver–insulin gate is associated with declining blood glucose control [9, 47, 49], the appropriate assessment of ICR after feeding has clear clinical significance.

To our knowledge, this is the first study to investigate the temporal clearance of insulin after a standardised mixed-macronutrient meal. By using a time-responsive model of ICR, our data provide new understanding into the hepatic extraction of insulin during physiological, non-steady-state conditions expected to occur in free-living. Altogether, we show that after ingestion of a mixed-macronutrient meal, ISR and ICR coordinate to produce a plasma insulin profile to maintain euglycaemia in normoglycaemic individuals with differing temporal profiles. In Lean-NGT individuals, there is a time course increase in ICR, thus constraining plasma insulin concentrations, and possibly in response to the complete postprandial suppression in glucagon, thereby minimising the risk of hypoglycaemia. In contrast, the adaptive glycaemic processes that involve ICR and ISR are dysregulated in type 2 diabetes. Our data stress the importance of taking into consideration differences in the secretion and clearance of insulin between groups when investigating the aetiology of postprandial hyperinsulinaemia in obese and type 2 diabetes populations.

## Supplementary Information

Below is the link to the electronic supplementary material.

ESM (PDF 167 KB)

## Data Availability

The raw data supporting the conclusions of this article will be made available upon reasonable request.

## Abbreviations

Adipo-IR: Adipocyte insulin resistance
AU: Arbitrary units
CEACAM1: Carcinoembryonic antigen-related cell adhesion molecule 1
GIP: Glucose-dependent insulinotropic polypeptide
GLP-1: Glucagon-like peptide 1
ICR: Insulin clearance rate
ISR: Pre-hepatic insulin secretion rate
Lean-NGT: Lean normoglycaemic
NGT: Normal glucose tolerance
Obese-NGT: Normoglycaemic with central obesity
PPAR-α: Peroxisome proliferator-activated receptor α
*Rd*_ins_: Insulin extraction rate
*S*_i_: Measure of insulin sensitivity
ɸ_total_: Measure of beta cell function
