# Supplementary material for: Elevations in plasma glucagon are associated with reduced insulin clearance after ingestion of a mixed-macronutrient meal in people with and without type 2 diabetes
Source: Diabetologia. 2024 Aug 13;67(11):2555–67. doi: 10.1007/s00125-024-06249-7 (PMC11519192; doi:10.1007/s00125-024-06249-7)
Supplement: Supplementary file 1 — ESM (PDF 167 KB) [file 125_2024_6249_MOESM1_ESM.pdf]

Elevations in plasma glucagon are associated with reduced insulin clearance after ingestion of a mixed-macronutrient meal in people with and without type 2 diabetes

Kieran Smith[1,2,3], Guy S. Taylor[2,3], Wouter Peeters[2,3], Mark Walker[4], Simone Perazzolo[5,6], Naeimeh Atabaki-Pasdar[1,7,8], Kelly A. Bowden Davies[2,9], Fredrik Karpe[1,6], Leanne Hodson[1,6], Emma J. Stevenson[2,3], Daniel J. West[2,3]

[1]Oxford Centre for Diabetes, Endocrinology and Metabolism, Churchill Hospital, University of Oxford, Oxford, UK

[2]Human Nutrition and Exercise Research Centre, Population Health Sciences Institute, Newcastle University, Newcastle upon Tyne, UK

[3]School of Biomedical, Nutritional, and Sport Sciences, Newcastle University, Newcastle upon Tyne, UK

[4]Translational and Clinical Research Institute, Newcastle University, Newcastle upon Tyne, UK

[5]Nanomath LLC, Spokane, WA, USA

[6]Department of Pharmaceutics, University of Washington, Seattle, WA, USA

[7]NIHR Oxford Biomedical Research Centre, Oxford University Hospital Trust, Oxford, UK

[8]Genetic and Molecular Epidemiology Unit, Lund University Diabetes Centre, Department of Clinical Science, Lund University, Malmö, Sweden

[9]Sport and Exercise Sciences, Manchester Metropolitan University, Manchester, UK

Electronic Supplementary Material

ESM Results

Due to evidence of multicollinearity between GLP-1 AUC_{240} and glucagon AUC_{240} in our original multivariate regression model, we performed a sensitivity analysis by substituting glucagon AUC_{240} with GLP-1 AUC_{240} (**ESM Table 2**). In an adjusted model that accounted for age, BMI, Adipo-IR and S_i, HOMA-IR and GLP-1 AUC_{240} were significant predictors to the overall variance in ICR AUC_{240} (adjusted R^2=0.632; p<0.001). These findings are akin to our results in **Table 3** showing that HOMA-IR and glucagon AUC_{240} contribute significantly to the prediction of ICR AUC_{240} (adjusted R^2=0.670; p<0.001).

ESM Table 1 Common indices of insulin sensitivity

Variable	Lean-NGT (*n*=12)	Obese-NGT (*n*=11)	Type 2 diabetes (*n*=19)	ANOVA *p* value	Lean-NGT vs Obese-NGT	Lean-NGT vs type 2 diabetes	Obese-NGT vs type 2 diabetes
Matsuda Index	12.9 (9.7, 17.4)	5.9 (3.9, 7.4)	3.3 (2.2, 5.7)	<0.001[a]	0.011	<0.001	0.567
OGIS Index	531.3 ± 39.9	473.7 ± 25.9	313.4 ± 62.8	<0.001	0.022	<0.001	<0.001

Data is presented as Means SD or Median (IQR)

[a] Data was analysed by a Kruskal Wallis H test

All *p*-values were adjusted for multiple comparisons

The Matsuda Index was calculated as 10,000/√[fasting plasma insulin x fasting blood glucose] x [average plasma insulin x average blood glucose], with glucose expressed mg/dl and insulin mU/l [1]

The Oral Glucose Insulin Sensitivity (OGIS) Index was determined using plasma insulin and blood glucose concentrations during the meal using the model described by Mari *et al* [2]

ESM Table 2 Independent predictors of insulin clearance from a multivariate linear regression model

Variable	log$_{10}$ ICR AUC$_{240}$								
	B	*95% CI B*		*SE*	*β*	*F*	*R^2*	*Adjusted R^2*	*p value*
		Lower	Upper						
Model 1						24.424	0.562	0.539	<0.001
Constant	0.305***	0.238	0.372	0.033					
log$_{10}$ HOMA-IR	-0.375***	-0.492	-0.259	0.057	-0.722				
S_i	0.000	0.000	0.000	0.000	0.095				
Model 2						16.901	0.653	0.614	<0.001
Constant	0.421***	0.308	0.534	0.056					
log$_{10}$ HOMA-IR	-0.213*	-0.380	-0.046	0.082	-0.410				
S_i	<0.000	0.000	0.000	0.000	-0.071				
Adipo-IR	<0.0001	0.000	0.000	0.000	-0.341				
GLP-1 AUC$_{240}$	-0.003*	-0.006	0.000	0.001	-0.230				
Model 3						13.315	0.655	0.606	<0.001
Constant	0.483***	0.224	0.743	0.128					
log$_{10}$ HOMA-IR	-0.196*	-0.376	-0.016	0.089	-0.377				
S_i	<0.000	0.000	0.000	0.000	-0.106				
Adipo-IR	<0.000	0.000	0.000	0.000	-0.330				
GLP-1 AUC$_{240}$	-0.003	-0.006	0.000	0.001	-0.232				
BMI	-0.002	-0.011	0.006	0.004	-0.085				
Model 4						12.454	0.687	0.632	<0.001
Constant	0.339*	0.042	0.635	0.146					

$\log_{10}$ HOMA-IR	-0.251**	-0.435	-0.067	0.091	-0.482
S_i	-0.010	0.000	0.000	0.000	-0.032
Adipo-IR	0.000	0.000	0.000	0.000	-0.271
GLP-1 AUC_{240}	-0.003*	-0.006	0.000	0.001	-0.258
BMI	-0.001	-0.010	0.007	0.004	-0.047
Age	0.003	0.000	0.007	0.002	0.202

Data were analysed by multivariate regression model on *n*=42

To meet assumption of linear residuals, the model included both transformed and non-transformed data

*** $p<0.001$

** $p<0.01$

*$p<0.05$

References

[1] Matsuda M, DeFronzo RA (1999) Insulin sensitivity indices obtained from oral glucose tolerance testing - Comparison with the euglycemic insulin clamp. Diabetes Care 22(9): 1462-1470. DOI 10.2337/diacare.22.9.1462

[2] Mari A, Pacini G, Murphy E, Ludvik B, Nolan JJ (2001) A model-based method for assessing insulin sensitivity from the oral glucose tolerance test. Diabetes Care 24(3): 539-548. 10.2337/diacare.24.3.539
